# Attainment of therapeutic vancomycin level within the first 24 h: Authors' response

**DOI:** 10.1186/s13054-019-2639-7

**Published:** 2019-11-13

**Authors:** João Pedro Baptista, Jason A. Roberts, Eduardo Sousa, Ricardo Freitas, Nuno Devesa, Jorge Pimentel

**Affiliations:** 1 Department of Intensive Care Medicine, Centro Hospitalar e Universitário de Coimbra, Coimbra, Portugal; 2 University of Queensland Centre for Clinical Research, Faculty of Medicine & Centre for Translational Anti-infective Pharmacodynamics, School of Pharmacy, The University of Queensland, Brisbane, Australia; 3 Departments of Pharmacy and Intensive Care Medicine, Royal Brisbane and Women’s Hospital, Brisbane, Australia; 4 Division of Anaesthesiology Critical Care Emergency and Pain Medicine, Nîmes University Hospital, University of Montpellier, Nîmes, France; 5 Emeritus Associated Professor on Intensive Care Medicine, Coimbra, Portugal

In sepsis, time is life. However, *time* is not the only variable of the complex equation of antibiotic therapy; the dose of antibiotic administered needs to be *adequate*. Subtherapeutic antibiotic concentrations potentially lead to decreased microbial killing, treatment failure, and emergence of resistance and/or increased mortality. Early therapeutic drug monitoring and timely dose optimization, ideally during the first 24 h, minimize the likelihood of subtherapeutic antibiotic concentrations and ineffective antibiotic therapy. Vancomycin remains a first-line option for the treatment of methicillin-resistant *Staphylococcus aureus* and other resistant Gram-positive bacteria. Of note, vancomycin is one of the antibiotics with the highest likelihood of under dosing [1]. Continuous infusion (CI), after adequate loading dose (LD), seems to have pharmacological advantages in the critically ill and enables more consistent achievement of therapeutic exposures.

A nomogram for dosing vancomycin can be easily used at the bedside of the patient, providing rapidly personalized dosing. One of the key factors facilitating the nomogram is the fact that renal clearance of vancomycin is strongly correlated with the measured urinary creatinine clearance (CL_CR_). Unfortunately, it is more common in clinical practice to use the less accurate mathematical estimates of renal function in unstable patients, instead of measured CL_CR_ [2]. Such an approach serves to compromise the reliability of the nomogram in the critical care setting.

We appreciate and read with interest the comments of Honoré et al., regarding our study in 2014, where we developed and validated a dosing nomogram for vancomycin in CI in a population of critically ill patients [3, 4]. Some clarifications, however, are needed. *First,* we never intended to compare CI with a LD of vancomycin; instead, we used, sequentially, LD (between 1 and 1.5 g) followed by CI (30 mg/kg/day). Later, with nomogram-guided dosing using an 8 h-CL_CR_, we achieved target vancomycin exposures in 84% of patients in the validation group in the first 24 h. Of these, 40% had demonstrated augmented renal clearance (8 h-CL_CR_ > 130 mL/min/1.73m^2^). *Secondly*, patients with compromised renal function or needing of renal replacement therapy were excluded in our study, meaning that our nomogram should not be considered applicable to this group of patients. *Third*, the volume of distribution and the half-life of vancomycin increases significantly in critically ill patients with renal insufficiency. On the other hand, vancomycin (medium molecular size molecule) is effectively cleared by continuous renal replacement therapies (CRRT). Considering the large inter-study variability, there is no clear recommendation about the optimal vancomycin regimen during CRRT [5]. A vancomycin loading dose of 15–20 mg/kg actual body weight would likely be more appropriate in CRRT patients. *Finally,* future studies confirming our dosing protocol are welcome; however, the chosen target population should be similar (with exclusion of patients under CRRT) so that the obtained results can be extrapolated to different contexts.

## Data Availability

Not applicable
